# Supplementary material for: Molecular Transmission Network of Newly Reported HIV Infections in Pengzhou, Sichuan Province: A Study Based on Genomics and Spatial Epidemiology
Source: Int J Environ Res Public Health. 2023 Jan 31;20(3):2523. doi: 10.3390/ijerph20032523 (PMC9915990; doi:10.3390/ijerph20032523)
Supplement: Supplementary file 1 [file ijerph-20-02523-s001.zip › ijerph-2089515-supplementary.pdf]

Table S1 Analysis of network entry rate and clustering results of molecules in each township

town	PLWH number	Number of entering the network	rate of network entry (%)	Number of clusters	rate of cluster (%)
Cifengzhen	12	1	8.33	1	1.85
Jiuchizhen	21	7	33.33	5	9.26
Sanjiezhen	14	5	35.71	3	5.56
Zhihezhen	42	18	42.86	9	16.67
Guihuazhen	13	6	46.15	4	7.41
Tianpengzhen	88	42	47.73	15	27.78
Mengyangzhen	50	24	48	13	24.07
Gexianshanzhen	21	11	52.38	5	9.26
Danjingshanzhen	11	6	54.55	5	9.26
Hongyanzhen	11	7	63.64	5	9.26
Junlezhen	11	7	63.64	4	7.41
Aopingzhen	25	16	64	12	22.22
Bailuzhen	3	2	66.67	1	1.85
Longfengzhen	27	18	66.67	11	20.37
Xiaoyudongzhen	9	6	66.67	3	5.56
Xinxingzhen	6	4	66.67	4	7.41
Shengpingzhen	20	14	70	6	11.11
Tongjizhen	19	14	73.68	7	12.96
Longmenshanzhen	4	3	75	2	3.7
Lichunzhen	54	41	75.93	12	22.22
